# The Nature and Treatment of Hepatic, Cardiac and Renal Dropsy

**Published:** 1905-01

**Authors:** A. M. Leonard


### SELECTIONS AND ABSTRACTS.

### The Nature and Treatment of Hepatic, Cardiac and Renal Dropsy.

By A. M. Leonard, M.D.

As pointed out some years ago by Pye-Smith, the diseases of one generation become the symptoms of the next. This is particularly true of paraplegia, amaurosis and dropsy. A hundred years ago the physician told the family of the patient that his "complaint had turned to dropsy," the effusion being the result of the relaxation of the fibres, the sinking of the animal spirits or the defluxion of a cold and moist humor.

Now, dropsy is only a step in the diagnosis, and no longer the culmination of the investigation.

The relation of Bright to his namesake is not generally understood. He did not discover that dropsy and albuminuria often go together, but he connected these symptoms with pathological changes in the kidneys.

We find the simplest expression of dropsy in the local form which results from the obstruction to the return of blood to the heart by pressure either on a finger, as when a ring is too small, or in a leg bandaged too tightly. The arteries are tougher and stronger, with higher blood-pressure, but the veins are yielding. Hence the blood gets in but can not get out. Undoubtedly the pressure on the lymphatic vessels helps in this result, but it is not necessary for its production, for it will follow venous thrombosis. The transudate is the water holding mineral salts and albumen in solution, together with minute portions of yellow pigment and other soluble extractives, the corpuscular part of the blood and the constituent essential for coagulation of fibrinogen does not transudate. This effusion is not produced by osmosis, for colloids like albumen and globulin would remain in the blood, while only crystalloids like chloride of sodium and sugar would pass out; besides the endosmotic current from the fluid of lower to that of higher specific gravity from the lymph-spaces to the blood-vessels would exceed the exosmotic from the blood to the lymph. In fact it is hard to see how the physical laws of diffusion under pressure agree with clinical facts, for experiment shows that the skin of frogs, and the veins of mammalia, while their epithelium is uninjured, offer far more resistance under pressure than do the dead animal membranes.

It is interesting to study the edema of the internal organs; for example, in the liver increased pressure in the portal blood stream comes from compression and occlusion of the trunk of the portal vein, or from increased pressure upon its capillary vessels; while in the spleen this venous congestion leads to swelling and hardening of the organ; in the gastric and less frequently in the intestinal mucous membrane, to hemorrhage; while serous exudation takes place from the visceral peritoneum. We are now able to trace cardiac dropsy to a mechanical origin. The pathology, like the physiology, of the heart and vessels, being largely that of a hydraulic machine, any constriction of a cardiac orifice, any defect in the valves, either from changes in their substance which prevent their closing or from dilatation of the orifice, which makes normal valves incompetent, produces congestion, fullness, over-pressure in the parts beyond the seat of disease, the anemia, with low pressure, in the parts in front, so the symptoms of cardiac disease can be divided into those of "venous congestion" and those of "arterial anemia." In uncomplicated aortic cases marked by pallor, syncope, breathlessness and liability to sudden death, the cause is arterial anema; venous congestion in primary mitral cases, in those of dilatation of the right cardiac chambers from chronic obstruction of the pulmonary circulation, in the latter stages of aortic disease when the mitral valve has given way, and in the last period of chronic Bright's where hypertrophy of the left ventricle is followed by dilatation and a small, hard, renal pulse becomes irregular and easily compressible.

The results of systemic venous congestion are twofold; in these cases in the mucous surfaces the distended venules give way, hemorrhage relieving the pressure. Thus, hematemesis may result by the same mechanism as in cases of cirrhotic portal congestion. Should hemorrhage not take place, an organ becomes swollen, dark-colored, firm, or even hard in texture, as may be seen in the spleen, the kidneys and the lungs from long-continued mitral obstruction. This form of dropsy strictly follows the law of gravity, for cardiac dropsy starts in the ankles, the venous congestion of the veins being favored by the weight of the blood. When a patient takes to his bed it affects his back, loins and upper extremities. One arm may be more swollen than the other if he habitually lies on one side.

Renal dropsy is of two separate pathological kinds. One variety occurs toward the end of the most chronic cases with insidious origin, atrophy of the renal cortex and cardiac vascular changes, mechanical in pathology but cardiac in origin. It appears when the pulse and other symptoms give evidence of cardiac failure. This form affects the feet first and the face but little, and it may be accompanied by extreme congestion or even hemorrhage in the lungs or stomach. But next there remains the much more striking, extensive and characteristic anasarca which marks both early and later stages of the more acute form of renal dieease, with definite onset and the well-marked characters of the urine which mark tubal or parenchymatous nephritis, anasarca in the strict sense of the word affecting all the lymph-spaces of the body at once, the integuments most rapidly and the serous membranes gradually—not governed by gravity, except slightly. It is distributed according to the location of distensible skin and loose connective tissue, such as the edematous conjunctiva, which produces the traditional "bright eye" and "the tear that does not fall," to the baggy lower eyelid or the often prodigious scrotal or preputial edema. This anasarca by mechanical distention of the skin and pressure on the vessels, aided by the anemia of the disease, produces that extreme and striking pallor which it is said Bright himself recognized as a symptom of tubal nephritis in the remark that "a large white man means a large white kidney." This is the clinical picture of the characteristic and purely renal form of dropsy which must be distinguished from the later mechanical dropsy of chronic Bright's disease. True renal anasarca can not be ascribed to a hydraulic origin, for, on this hypothesis, there is no valid physiological cause for its occurrence. Its distribution would be different, and as a matter of fact it does not occur in other diseases when hydraulic conditions are favorable. Distinctive renal anasarca is certainly not the mere result of scanty secretion of urine, for it often remains even after the kidneys have retained their normal excretion of water; nor does it accompany great diminution or even suppression of urine, due to other causes than nephritis. No anatomical evidence has yet been offered to support Cohnheim's hypothesis of a change in the walls of the capillary vessels favoring the escape of serum. Nor does any change in the constitution of the blood, the diminution of albumen and other colloids, the diminution of red corpuscles, or the excess of urea, explain renal dropsy, since we find nothing like it when anemia or albuminuria or uremia is present from other causes. Renal dropsy in its more acute and characteristic form is not a passive exudation, but inflammatory. This is the only satisfactory hypothesis, for, in the first place, this agrees with its universal distribution and comparative independence of gravity, while it explains its absence in the more chronic and least inflammatory form of kidney disease. It is difficult to draw the line between hydrothorax or ascites in cases of Bright's disease and pleurisy or peritonitis with effusion, and it is far harder than in cases of cardiac dropsy. Should the serous exudation be inflammatory we need not need artificial distinctions, for lymph, serum or occasionally even pus are alike the exudation of renal peritonitis, pericarditis or pleurisy.

Again, we find that edema occurs in cases of phthisis, in anemia, in scurvy, and moderate edema is often seen during convalescence from fevers or other acute diseases, disappearing after convalescence. There may be evidence of dilatat ion on the right side of the heart and general venous congestion, as in other examples of chronic obstruction in pulmonary capillaries. Should the dropsy follow acute diseases, the pulse is weak and compressible while the patient is liable to giddiness or even syncope. Here edema of the ankles depends upon a weak left ventricle causing arterial blood pressure, with stagnation in the veins, particularly where the circulation has gravity against it; also, the cardiac muscles suffer in the same way as do the muscles of the limbs, and the circulation is weak for the same reason as the patient's grasp is feeble and his gate unsteady. The edema which occurs in various forms of anemia has been ascribed to the watery state of the blood, alleging that it is more diffused through the vascular walls; but there is no evidence of this, and exosmosis would be diminished, not increased, by a fall in the specific gravity of the contained fluid. The dropsy of anemia is due to the same cause as stated above—weakness of the cardiac muscle and consequent low pressure in the arteries.

In diagnosis of these forms of dropsy we know that edema confined to one limb depends upon external pressure upon the corresponding venous trunk or upon the formation of thrombus within it, such as the edema of an arm from the pressure of an aneurism, an abscess or malignant growth on the axillary vein, edema in a leg from thrombosis after fever or parturition, such as seen in milk-leg. When it comes to the diagnosis of portal dropsy, pathologically as local as the cases first mentioned, the decision rests upon its limitation to the parts below the diaphragm. When we see a patient with emaciated face and arms, but with a big belly and swollen legs, we conclude at once that disease of the liver has caused first ascites and secondly edema of the parts supplying the inferior cava. Here the genital organs escape from dropsy, and so does the abdominal wall—a valuable diagnostic point between hepatic ascites and that due to renal disease. The patient may say that the legs began to swell before the belly, but apart from wilful deception, this depends upon the tight shoe and puffy ankle observed when the patient retires; which early ascites is naturally attributed by middle-aged men and women to fat or flatulence. It is hard to detect a small amount of ascites—more than a pint of serum may be present without causing demonstrable dullness in the flanks, even when we contrast the results of percussion in the right and left lateral position—but the abdomen becomes speedily more rounded and uniform in outline and the umbilicus begins to flatten out, in striking contrast to its deep depression in obesity. A common trouble is to distinguish ascites from inflammatory effusion into the peritoneal cavity, which is often painless, even when secondary to cancer or tubercular growth.

Here, in these cases, physical signs are exactly the same as in ascites from portal obstruction, and the diagnosis must rest upon the presence or absence of other symptoms pointing to the liver and to the possibility of the existence of cancer, of tubercle, or of cirrhosis. In general, dropsy which is of cardiac origin, whether primary or secondary, by its close dependence upon gravity, is the cardinal point. The ankles and instep swell first, and the edema which was noticed at night disappears in the morning; or, again, a patient may notice while dressing a little puffiness in the face, which disappears in an hour or two, being rarely seen by the physician. Swelling of the arms distinguishes cardiac from hepatic dropsy, and if sometimes one and sometimes the other is far more swollen than its fellow or than the face, we may be almost certain that dropsy is not of the renal origin. In the chronic anasarca of heart disease the face is almost exempt, and if the genital organs are swollen at all it is but little. The sallow tint or actual jaundice produced by a nutmeg liver completes the picture of advanced mitral dropsy, but jaundice is also common in dropsy from cirrhosis. It is seldom seen when the cardiac failure is secondary to pulmonary or systemic obstruction from chronic bronchitis or the most chronic form of renal disease. General dropsy of direct and immediate renal origin is recognized by its rapid onset, by the marked pallor which accompanies it and by its local distribution, for, while the anasarca is universal, the legs suffer as in all kinds of general dropsy, the face, the loins, and the genital organs, which are seldom or never greatly swollen in cases of cardiac origin, and never in those due to portal obstruction, are quickly aud invariably seats of renal anasarca. The puffy eyelid, the white, glistening, watery conjunctiva, the "lumbar cushion," the scrotum swollen to the size of a child's head, and the penis distended like a bladder, translucent—these features are striking symptoms of tubal nephritis. Yet, however, great the preputial edema, there is never retention of urine. The serous cavities probably never entirely escape, and often we see ascites and effusion in one or the other pleura almost from the first. Fluctuation is more difficult to obtain than in cases of cardiac or nephritic dropsy.

When we come to the treatment of dropsy, naturally it depends upon an accurate diagnosis of its nature and origin. Should slight edema of the lower extremities, as the result of functionally weak circulation, be seen, there is no direct object in removing it, yet its disappearance is a comfort to the patient. Where there is more extensive dropsical effusion, treatment must be prompt and vigorous if it is to be efficient. First object, get rid of the effused serum. Do this mechanically by puncture of the legs or by tapping the abdomen or the chest, or physiologically by increasing the natural output of water from the body. Water leaves the body by four channels—by the kidneys, the skin, the lungs and the bowels. It is in health that the last amount is inconsiderable, while the cutaneous and renal excretions alternate, the healthy kidney secreting more freely when the skin is cold and dry, and much less so when there is abundant excretion of sweat. The excretion of water by the lungs is more constant than that by the other two chief exits, being increased where exercise causes deep and rapid breathing. Unfortunately, in disease we can not avail ourselves of the lungs to increase the output of water, but we can so powerfully stimulate the intestinal secretion that purging becomes as important a means of elimination as diuresis or sweating. These three methods of unloading the body of its excess of water exist, and we must select the best one suited for our purpose. For example: Purging, when efficient, is the most certain and the most speedy in its action—indeed, scarcely inferior to direct removal of water by mechanical means. Hydragogue purgatives are more sure in their effects than drugs which act upon the kidneys or the skin, but the objection to this treatment is the danger of syncope from the rapid withdrawal of too much fluid and the subsequent fall of blood-pressure. Hence it is wise to refrain from purging in cases of cardiac dropsy, while in acute nephritis it finds its most appropriate place, especially in children after scarlet fever. In more chronic cases of Bright's disease one must use purgatives with more caution, since there is a tendency to catarrhal colitis.

We do not want to produce intestinal irritation, and attempts at hard purging in the later stages of renal disease may lead to frequent small evacuations, chiefly of mucous and blood, accompanied by tenesmus, pain and exhaustion, with but little fluid. Diaphoretics, unfortunately, are more uncertain drugs than purgatives, and some of the most efficient, such as antimony, are contraindicated by their other results. The old-fashioned rule was that salts of potash are most suitable for the kidneys, those of soda for the liver, and ammoniacal salts for the skin; hence, we can give a solution of acetate of ammonia in cases of anasarca, and if the skin be kept warm it may act nicely. More efficient to produce sweating is the hot-air bath, but this powerful remedy must be used with caution, especially at first. Should the skin fail to act, it is intolerably distressing to the patient, and may be followed by severe uremic symptoms, due to dislodgement of some retained urinary poison from the tissues, along with their lymph and its free passage into the circulation. The patient may be put in a hot-water bath, the temperature to be increased instead of allowing it to cool, staying in for a quarter of an hour or twenty minutes. He should be quickly dried and wrapped in a blanket. A free and not ungrateful perspiration follows, and when this is over he should be well rubbed and put to bed. Jaborandi, or its alkaloid, pilocarpine, is an efficient diaphoretic, but is an emetic and therefore less applicable for renal dropsy in itself than for cases of dropsy complicated by headache and the other symptoms of uremia. In renal dropsy it is unwise to use the kidneys as a channel of elimination. Should the dropsy be severe, the kidneys are in an inflamed condition with the blood-pressure already high and the excretory tubes blocked. Prescribe here a diuretic mixture and you will be struck by its inefficiency. You dare not use such stimulating drugs as squills, broom or copaiba, nor the vascular diuretics of which digitalis is the chief, but either be content with a little acetate or iodide of potassium, or, what is the better course, to give the diseased organ as much rest as possible. In cases of acute nephritis, with the urine scanty or suppressed, drinking plenty of water will dilute the blood and the urine without causing any irritation, and a patient in this condition should therefore be encouraged to drink freely. In cases of hepatic and cardiac dropsy where the kidneys are healthy, diuretics are, on the other hand, the safest and most efficient mode of treatment. In the most common forms of ascites, that due to cirrhosis, the case often comes under notice when the abdomen is already full and when swollen legs and scanty urine show that there is considerable pressure on the renal and iliac veins. Then tap at once, or diuretics will fail; but after the pressure is relieved the kidneys naturally secrete more freely. Then is the time to help them. An old-fashioned mixture of acetate of potassium, sweet spirits of nitre, tincture of squills and decoction of broom-tops has been long a favorite compound in many hands, while another very valuable mixture is that of resin of copaiba. A third most efficient and far from unpleasant remedy is the English "haustus imperialis" (lemonade, with cream of tartar and a little sugar), which is a gentle laxative as well as a powerful diuretic.

In cardiac dropsy purging must be cautiously tried, and diaphoresis is difficult to obtain, and often does more harm than good. Here depend almost exclusively upon diuretics. In addition to those already mentioned digitalis is an invaluable means, not only of regulating the heart, but also of increasing diuresis by inducing rise of blood-pressure in the renal arteries. Dropsy due to cardiac failure must be treated by our recognition of its pathology, and in practice they are best met by the exhibition of iron, with which we may often advantageously combine digitalis by more meat in the diet, by alcohol taken with the meals, and by as much exercise as the patient's strength will admit. In the secondary dropsy, hydraulic in nature, occurring in the latter stages of Bright's disease, when the hypertrophied heart has become dilated and the hard pulse has accordingly become compressible and irregular, the condition approaches that of primary mitral insufficiency with failing compensation, and then digitalis is as useful as it is injurious with a high blood-pressure, hypertrophied ventricle and incompressible pulse. Often we can not wait for the action of diuretics, purgatives or diaphoretics, for the lungs are oppressed, the movements of the diaphragm are impeded by the unyielding mass of water in the belly, and the kidneys can scarcely secrete owing to the high venous and low arterial pressure, due perhaps not only to the embarrassed action of the heart, but also to direct compression of the renal veins and inferior cava by the effusion. Should there be universal dropsy it is best to tap the abdomen, for relief is thus rapidly obtained. If there be hydrothorax of one side and edema of the lungs or only slight effusion on the other, the chest should be aspired. When the case is less urgent the patient should be made to sit up until his legs are full of serum, and they should then be punctured with a needle, or, more painlessly and efficiently, with the point of a sharp and clean lancet, which should penetrate the skin so as to reach the lymph-spaces of the subcutaneous fascia, not drawing blood. Or, two or three larger incisions may be made. The danger of the two latter methods is of exciting troublesome chronic sores, or even erysipelas or gangrene, unless done antiseptically. With acupuncture carefully performed, there should not be any such result except, perhaps, in cases of renal dropsy. Wash the legs with hot soap and water, then anoint with olive oil or cold cream or benzoated lard, which I have found more effectual in preventing dermatitis than zinc ointment, and after the acupuncture has been rapidly and efficiently performed from the instep to the knee the legs should be wrapped in warm blankets and allowed to drain as freely as possible. Similar punctures may be made in the scrotum with the same precautions against inflammation from the salt serum running over the skin. Employ paracentesis in most cases of considerable hepatic dropsy, and after its performance use diuretics with good success until obliged to tap again. In cases of renal dropsy you may be obliged to tap the chest and on rare occasions the pericardium, but avoid paracentesis of the abdomen as long as possible for fear of exciting peritonitis. Acupuncture is useful, but the greatest care must be taken to avoid subsequent inflammation. Cardiac dropsy may require tapping for ascites, but very seldom for hydrothorax, but the more satisfactory treatment is by acupuncture. Get your patient out of bed and let him sit in an old-fashioned easy chair—if possible—with high elbows, plenty of cushions and head-rests at the side. A night so spent by the fire will enable him to sleep as he has not for many days in bed, while the never-ceasing action of gravity draws the water into his legs. Next day skilful acupuncture will produce such a flow of serum that in certain cases the chest and belly will quickly be relieved and the kidneys will begin to secrete more freely. Then is the time to prescribe your medicines, and the results are often astonishing. A fellow creature waterlogged and breathless, in the most urgent distress, is so changed that his limbs and body are no longer swollen, his breathing is tranquil, he can talk without difficulty, and regains his appetite. With the aid of digitalis and extreme care in living he may maintain a state of convalescence for a long period, if possible for years. Stimulation can only be exerted upon active tissues, upon leucocytes, glandular epithelium, muscular fibres, ganglion cells or nerve fibres, but the process of absorption has been hitherto supposed to be purely mechanical. It depends on the difference in pressure between the great venous trunks and the lymph-spaces, and is therefore dependent upon the activity of the circulation; or, in other words, the excess of the arterial blood-pressure over that in the great veins. This view is entirely in accordance with experience of the action of digitalis, iron and other cardiac remedies on the one hand, and of diuretics, purgatives and diaphoretics on the other.—*The Medical Times.*

